# Ginsenoside Rb1 as an Anti-Diabetic Agent and Its Underlying Mechanism Analysis

**DOI:** 10.3390/cells8030204

**Published:** 2019-02-28

**Authors:** Ping Zhou, Weijie Xie, Shuaibing He, Yifan Sun, Xiangbao Meng, Guibo Sun, Xiaobo Sun

**Affiliations:** 1 Institute of Medicinal Plant Development, Peking Union Medical College and Chinese Academy of Medical Sciences, Beijing 100193, China; zhoup0520@163.com (P.Z.); ginseng123@163.com (W.X.); wym91116@163.com (S.H.); xbmeng@implad.ac.cn (X.M.); 2 Beijing Key Laboratory of Innovative Drug Discovery of Traditional Chinese Medicine (Natural Medicine) and Translational Medicine, Institute of Medicinal Plant Development, Peking Union Medical College and Chinese Academy of Medical Sciences, Beijing 100193, China; 3 Key Laboratory of Bioactive Substances and Resource Utilization of Chinese Herbal Medicine, Ministry of Education, Beijing 100193, China; 4 Key Laboratory of Efficacy Evaluation of Chinese Medicine against Glycolipid Metabolic Disorders, State Administration of Traditional Chinese Medicine, Beijing 100193, China; 5 Institute of Medical Information, Chinese Academy of Medical Sciences, Beijing 100020, China; sun.yifan@imicams.ac.cn

**Keywords:** ginsenoside Rb1, diabetes, diabetic complication, multi-target effects

## Abstract

*Panax ginseng* and *Panax notoginseng*, two well-known medical plants with economic value, have a long history of use for managing various diseases in Asian countries. Accumulating clinical and experimental evidence suggests that notoginsenosides and ginsenosides, which are the major bioactive components of the plants, have a variety of beneficial effects on several types of disease, including metabolic, vascular, and central nervous system disease. Considerable attention has been focused on ginsenoside Rb1 derived from their common ownership as an anti-diabetic agent that can attenuate insulin resistance and various complications. Particularly, in vitro and in vivo models have suggested that ginsenoside Rb1 exerts various pharmacological effects on metabolic disorders, including attenuation of glycemia, hypertension, and hyperlipidemia, which depend on the modulation of oxidative stress, inflammatory response, autophagy, and anti-apoptosis effects. Regulation of these pathophysiological mechanisms can improve blood glucose and insulin resistance and protect against macrovascular/microvascular related complications. This review summarizes the pharmacological effects and mechanisms of action of ginsenoside Rb1 in the management of diabetes or diabetic complications. Moreover, a multi-target effect and mechanism analysis of its antidiabetic actions were performed to provide a theoretical basis for further pharmacological studies and new drug development for clinical treatment of type 2 diabetes. In conclusion, ginsenoside Rb1 exerts significant anti-obesity, anti-hyperglycemic, and anti-diabetic effects by regulating the effects of glycolipid metabolism and improving insulin and leptin sensitivities. All of these findings suggest ginsenoside Rb1 exerts protective effects on diabetes and diabetic complications by the regulation of mitochondrial energy metabolism, improving insulin resistance and alleviating the occurrence complications, which should be further explored. Hence, ginsenoside Rb1 may be developed as a potential anti-obesity, anti-hyperglycemic, and anti-diabetic agent with multi-target effects.

## 1. Introduction

Diabetes mellitus (DM) is a metabolic disorder characterized by hyperglycemia caused by insufficient insulin secretion and/or insulin action. The increased prevalence of DM, especially type 2 diabetes (T2DM) [1,2,3], is a multi-factor disease resulting from both genetic factors, such as obesity, impaired postprandial insulin secretion, and partial pancreatic ß-cell damage, and environmental factors, including obesity, unhealthy dietary patterns, lack of exercise, and aging [4]. These factors lead to persistent hyperglycemia and a subsequent decrease in insulin sensitivity, which in turn causes a series of metabolic disorders [5]. Moreover, sustained glucose and lipid metabolism disorder will lead to various microvascular and macrovascular complications, such as stroke, ischemic heart disease, diabetic nephropathy (DN), cognitive dysfunction, and retinopathy [6]. These complications caused by diabetes impose ever-increasing burdens on healthcare systems in developed as well as developing countries. Diabetic macroangiopathy refers to cardiovascular and cerebrovascular diseases, with atherosclerosis as the main manifestation. Diabetic microangiopathy is mainly characterized as the thickening of the basement membrane and the deposition of transparent substances in the retina, kidney, nerve tissue, and elsewhere.

Recently, there has been a shift in the drugs prescribed for T2DM from agents that stimulate insulin secretion, such as sulfonylureas, towards agents that increase insulin sensitivity, such as biguanides (BGs) [7] and thiazolidinediones (SUs) [8,9,10]. These latter classes of drugs activate the hepatic adenosine monophosphate-activated protein kinase (AMPK) [11,12,13], phosphatidylinositol 3-kinase (PI3K), and protein kinase B (PKB/Akt) [14,15,16], stimulate fatty acid oxidation in an AMPK- and peroxisome proliferator activated receptor-α (PPAR-α)-dependent manner, and inhibit the interference with c-Jun amino-terminal kinases (JNKs) and insulin action activated by inflammatory cytokines and free fatty acids [17,18], all of which are new targets and new ways of reducing blood sugar, obesity, and diabetes symptoms.

Glucose transporter type 4 (GLUT4) also plays an important role in maintaining blood glucose homeostasis and its presence can reduce insulin resistance. The primary function of GLUT4 is partial glucose transport, which is mediated by the PI3K and Akt signaling pathways. In addition, many mechanisms initiate and sustain damage to the vasculature, including the classical polyol pathway, glycosylation end products pathway, protein kinase C pathway, and hexosamine pathway [19,20].

Conventional diabetes treatments include SUs, BGs, TZDs [21], and alpha-glucosidase inhibitors [22,23]. These drugs usually have considerable side effects, such as hypoglycemia, drug resistance, dropsy, and weight gain. With advancing research on diabetes, treatment has progressed from only increasing insulin’s hypoglycemic effect to controlling glucose metabolism, enhancing insulin receptor sensitivity, inhibiting insulin resistance, regulating protein non-enzymatic glycosylation, and reducing fatty acid metabolism and other aspects [24,25]. Although numerous preventive and therapeutic strategies and drugs for diabetes have been developed, the treatment and management of diabetes remain highly unsatisfactory. Therapies currently used to control and treat diabetes and its complications rely primarily on chemical or biodegradable drugs. What is more serious is that there is also a growing phenomenon of diabetic complications [26]. Therefore, there is an urgent need to develop new, better, and safe natural hypoglycemic agents as alternatives for managing diabetes and its complications.

Traditional Chinese medicines and their natural active ingredients have a variety of hypoglycemic effects and effectively control and delay the development of diabetes and complications by scavenging oxygen free radicals, improving blood hypercoagulability, inhibiting protein non-enzymatic glycation, inhibiting aldose reductase, correcting fat and protein metabolism disorders, and inhibiting platelet aggregation [27,28,29,30,31]. *Panax notoginseng* (Burk.) F.H.Chen [32] and *Panax ginseng* C.A.Mey [33] (Araliaceae) are commonly used in Chinese medicine and the roots have been used for the treatment of hemoptysis, hemostasis, and hematoma for several hundred years in China and other Asian countries due to their cardiovascular effects [33,34,35], including in diabetes and its complications [36,37,38]. Pharmacological studies have shown that *P. notoginseng* and its extracts have many functions, such as anti-inflammation [39,40,41,42], anti-oxidation, platelet aggregation inhibition, blood glucose regulation [43,44] and blood pressure regulation [45,46,47], insulin resistance improvement [31,48], neuronal apoptosis inhibition [49,50,51], and neuronal protection [52,53,54,55]. In particular, the promising role of ginsenoside Rb1, the main protopanaxadiol-type ginsenoside isolated from *P. notoginseng* and *P. ginseng*, as an anti-diabetic is widely publicized [56,57,58,59,60,61,62,63,64]. Ginsenoside Rb1 possesses anti-diabetic and insulin-sensitizing properties [56,59].

However, to date, no systematic review has been conducted to assess the protective effects and underlying mechanisms of how ginsenoside Rb1 combats diabetes and its complications. If a systematic review and analysis of preclinical studies of ginsenoside Rb1 against diabetes and its complications were to be carried out, all evidence available from animal experiments or preceding clinical trials would provide a theoretical basis for further pharmacological studies and new drug development for clinical treatment of T2MD. 

## 2. Source and Chemistry

**Figure 1:**
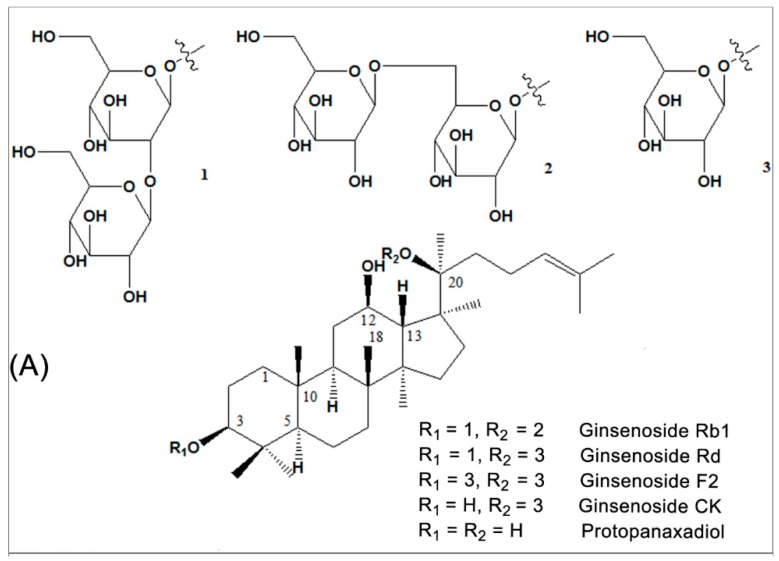

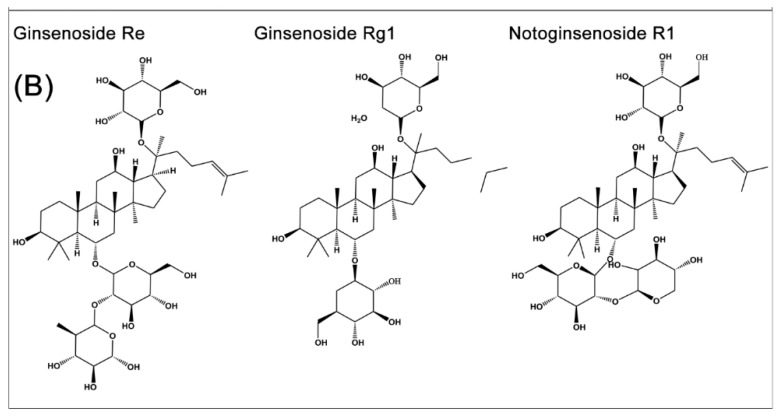
Chemical structural formula of the representative saponin components of *Panax notoginseng and Panax ginseng*. (**A**) Component structure of ginsenoside Rb1 and its metabolites; (**B**) ginsenoside Re, Rg1 and notoginsenoside R1.

*P. notoginseng and P. ginseng*, two of the most widely used traditional Chinese herbs, have an approximately 2500-year medicinal history in Eastern Asia. It is a slow-growing perennial plant that belongs to the genus *Panax* in the Araliaceae family [65]. The vast array of body systems treated by *P. notoginseng* and *P. ginseng* include the immune system, the nervous system, the cardiovascular system, and blood circulation [33,35]. Saponins are one of the main kinds of active ingredient of *P. notoginseng* [32,66] and *P. ginseng* [33,35]. So far, more than 70 kinds of monomeric saponins have been isolated from different parts of *P. notoginseng* (roots, stems, leaves, flower buds, and seeds.) [32,66] and most of these monomeric saponins are separable [33,35]. These consist of the dammarane type of 20 (S)-protopanaxadiol and 20(S)-protopanaxatriol saponins. Among them, ginsenoside Rb1 (Figure 1A) is the main protopanaxadiol saponin and ginsenoside Re, Rg1 and notoginsenoside R1 (Figure 1B) are the main representative protopanaxatriol components. These four components, shown in Figure 1B, are part of the total saponins of *P. notoginseng*. All ginsenosides have the same four-ring hydrophobic structure, but different numbers of sugar moieties. Ginsenoside Rb1 and Rg1 are the most abundant in *P. notoginseng* and the quality standard is also based on the total amount of ginsenoside Rb1, Rg1 and notoginsenoside R1, which is not less than 5.0% as the standard for measuring the *P. notoginseng* quality. Ginsenoside Rb1, the most important active factor [67], is also isolated from other *Panax* species, including *Panax quinquefolius* (American ginseng) and *Panax ginseng* (Asian ginseng). It is thought that the variability of the sugar component may be associated with the specific action of each ginsenoside [68]. More importantly, Rb1 shows certain pharmacological effects in maintaining blood circulation, improving myocardial ischemia, anti-arrhythmia, anti-shock, anti-diabetic, improving intelligence, anti-aging, anti-oxidation, anti-cell proliferation, and anti-tumor [32,33,35].

## 3. Anti-Diabetic Effects and Mechanisms of Ginsenoside Rb1

Insulin resistance is an abnormal physiological state and the main cause of type 2 diabetes (insulin-independent diabetes), meaning that target cells are not sensitive to insulin and require relatively more insulin to maintain normal blood glucose levels, which can be caused by abnormal structure of insulin, the abnormal binding of insulin receptors, insulin degradation, insulin receptor defects, metabolic signal transmission error, etc. Recently, the tendency of drug prescription for type 2 diabetics has moved from agents that stimulate insulin secretion toward agents that increase insulin sensitivity by activating the hepatic AMPK, PI3K, and Akt activity [12,14,15,16,69,70]. 

The increased prevalence of obesity has focused attention on a worldwide problem that is not one of famine or infection, but one of surplus. Increasing research results show ginseng and ginsenoside Rb1 possess anti-obesity [71], anti-hyperglycemic [72,73], insulin-sensitizing [74], and anti-diabetic properties [75,76], which may be mainly realized by improving glucose tolerance, reducing hepatic fat accumulation [59,64], increasing the sensitivity of insulin, exhibiting adjuvant treatment, suppressing adipocyte lipolysis [77], and regulating adipocyte development and functions [64,73,75]. These effects contribute to the treatment of diabetes and delay in the development and progression of diabetic complications. 

### 3.1. In Vivo

Current in vivo results (Table 1) have shown that four-week intraperitoneal administration of ginsenoside Rb1 could significantly reduce food intake, body weight gain, and body fat content and increase energy expenditure in high-fat diet (HFD)-induced obese mice and rats (adult male Long–Evans rats) (*p* < 0.01) [62,71]. Meanwhile, Rb1 may obviously reduce hepatic fat accumulation, suppress adipocyte lipolysis in obese and diabetic mice (*p* < 0.01) [64,78], and decrease fasting blood glucose (FBG) [78,79]. All of these contribute to improving glucose tolerance and the sensitivity of insulin, as shown in Table 1.

First, ginsenoside Rb1, which was injected intraperitoneally (60 mg/kg body mass, daily) for 12 d, decreased body weight growth gain, food consumption, adipose tissue, and leptin levels in KK-Ay DM mice (*p* < 0.01) [73]. Ginsenoside Rb1 ameliorated the HFD-induced increase in FBG, impaired glucose tolerance, and reduced insulin sensitivity to exert its anti-diabetes effects in a HFD-induced mouse model for T2DM (*p* < 0.01) [79]. 

Second, treatment with ginsenoside Rb1 (20 mg/kg body mass, daily) for 14 d alleviated hepatic fat accumulation and increased insulin sensitivity; these effects were accompanied by reduced liver weight and hepatic triglyceride content in obese diabetic *db/db* mice (*p* < 0.01) [64] and upregulated perilipin in 3T3-L1 adipocytes [64]. Treatment with ginsenoside Rb1 by intraperitoneal injection (20 mg/kg body mass, daily) for 14 d also significantly reduced the homeostasis model assessment-insulin resistance and elevated glucose consumption of 3T3-L1 adipocytes; FBG and fasting insulin showed a declining trend in male *db/db* mice [78]. The mechanisms may be closely related to activating the insulin signaling pathway (Akt), promoting translocation of GLUTs to increase glucose uptake in adipocytes [78]. 

Third, intraperitoneal injections of Rb1 (10 mg/kg body mass, daily) for both 21 d [62] and 4 weeks [71] significantly reduced body weight gain, improved glucose tolerance, homeostasis model assessment, and fasting plasm insulin in HFD-induced obese mice and rats compared to vehicle treatment (*p* < 0.01) [62,71]. Importantly, treatment with ginsenoside Rb1 also reduced pro-inflammatory cytokines (TNF-α, IL-6, and/or IL-1β) in adipose tissue and the liver, which are involved in regulation of the NF-κB pathway (p-IKK and p-IκBα), leptin p-STAT3 signaling, and negative regulators of leptin signaling (SOCS3 and PTP1B) [62]. These effects may improve insulin sensitivity by regulating glucose and lipid metabolism against obesity and diabetes.

Generally, the underlying molecular mechanism is directly bound up with regulating glycolipid metabolism in muscle, liver, and adipose tissues. Ginsenoside Rb1 may regulate glucose and lipid metabolism, improve glucose tolerance and insulin sensitivity, and reduce inflammatory cytokines and the inflammatory response in adipose tissue and the liver, which is involved in regulation of the NF-κB pathway.

### 3.2. In Vitro

In vitro experimental studies also indicate that ginsenoside Rb1 possesses insulin-sensitizing activity and regulates the effects of glucose metabolism; thus, this compound may be developed as a potential anti-diabetic drug [61,74,75,76], as shown in Table 1. 

Adipocyte cell lines derived from mice, especially 3T3-L1 cells, are extensively used in adipose tissue metabolism studies [80,81]. It was proved that Rb1 significantly stimulated basal and insulin-mediated glucose uptake in a time- and dose-dependent manner in 3T3-L1 adipocytes [75], promoted GLUT1 and GLUT4 translocations to the cell surface via increasing the phosphorylation of insulin receptor substrate-1 and protein kinase B, and stimulated PI3K activity [75]. In addition, 10 μM Rb1 increased lipid accumulation by approximately 56% [74] and ginsenoside Rb1 facilitated adipogenesis of 3T3-L1 preadipocytes in a dose-dependent manner. Several processes are involved: Rb1 inhibited the proliferation of preconfluent 3T3-L1 preadipocytes, increased the mRNA and protein expression of the peroxisome proliferator-activated receptor γ (PPARγ2) and recombinant human CCAAT/enhancer binding protein alpha (C/EBPα), as well as the mRNA of ap2, and Rb1 may be involved in the process of adipose, glucose, and lipid metabolism in differentiating adipocytes (*p* < 0.05) [74]. 

In C2C12 myotubes, incubation with ginsenoside Rb1 enhanced basal AdipoR1 and AdipoR2 expression in a time- (1–12 h) and dose-dependent (Rb1, 0.001–100 µM) manner [61]. In C2C12 myotubes and 3T3-L1 cells, Rb1 induced GLUT4 translocation partly by activating the adiponectin signaling pathway [61,75].

Furthermore, ginsenoside Rb1 acts as an insulin sensitizer and thus has a hypoglycemic function [82,83]. It activates AMPK [82,83], an important protein associated with energy metabolism, regulates energy metabolism, and promotes glucose uptake by at least partially activating the insulin signaling pathway [82]. 

**Figure 2:**
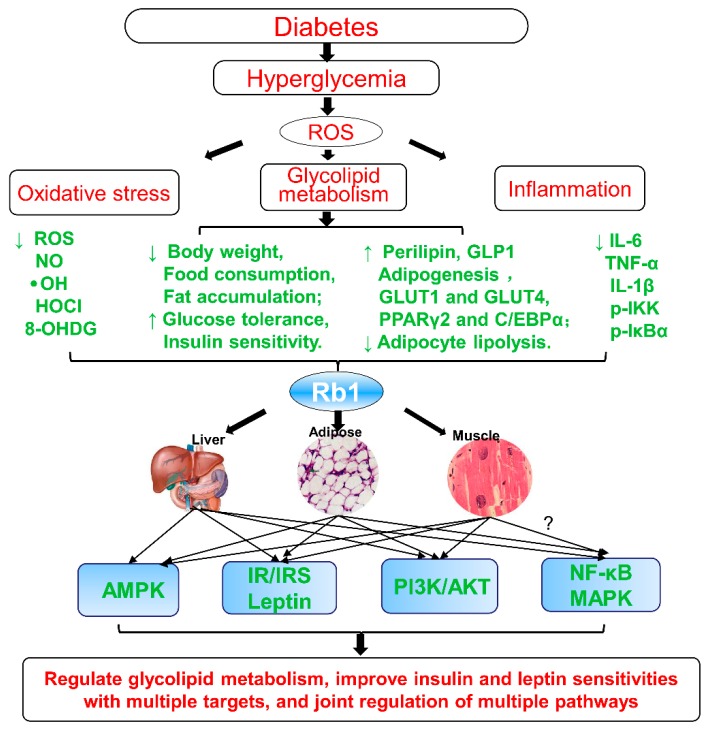
Summary and functional network target analysis of ginsenoside Rb1, which exerts significantly anti-obesity, anti-hyperglycemic, and anti-diabetic effects on diabetes via multiple links across regulatory mechanisms and multi-target effects. HOCl, hypochlorous acid; (IR), insulin signaling pathway; **·**OH, hydroxyl radical; ROS, reactive oxygen species; (↓), downregulation or inhibition; (↑), upregulation or activation; (?), uncertainty or undetermined.

In summary, as shown in Figure 2, Rb1 could promote adipogenesis, enhance the GLUT1 and GLUT4 translocation, upregulate the perilipin levels in adipocytes, regulate the glycolipid metabolism, and improve insulin and leptin sensitivities. All of these effects are closely associated with reducing hepatic fat accumulation, suppressing adipocyte lipolysis, decreasing 11β-hydroxysteroid dehydrogenase type I (11β-HSD1) in the liver and adipose tissues, regulating energy metabolism, increasing adiponectin receptor expression, upregulating perilipin in adipocytes [79], improving PPARγ2 and C/EBPα, activating the adiponectin signaling pathway [74], regulating AMPK, PI3K, and Akt signaling pathways [75,82], inhibiting high glucose (HG)-induced caspase-3 and apoptosis [84], negatively regulating the leptin signaling [62,79], and regulating NF-κB and leptin–STAT3 signaling pathways, at least partially, via decrease of inflammatory markers [85]. Hence, ginsenoside Rb1 may be developed as a potential anti-obesity, anti-hyperglycemic, and anti-diabetic agent, as shown in Table 1.

Based on the above summary and analysis, ginsenoside Rb1 exerts significant anti-obesity, anti-hyperglycemic, and anti-diabetic effects by regulating the effects of glycolipid metabolism and improving insulin and leptin sensitivities, as showed in Figure 2. These effects show the characteristics of multiple targets and joint regulation of multiple pathways:

- Inhibit lipolysis and reduce free fats release and ectopic triglycerides deposition;
- Reduce hepatic fat accumulation and suppress adipocyte lipolysis;
- Decrease inflammatory stress in adipose and liver tissues;
- Promote adipogenesis and the 3T3-L1 adipocytes differentiation;
- Upregulate PPARγ and enhance GLUTs expression and translocation of GLUT1/ GLUT4;
- Upregulate perilipin in adipocytes and in fat cells;
- Decrease 11β-HSD1 and its mRNA in liver and adipose tissue;
- Decrease the expression of inflammatory markers (IL-6, IL-1β, and p-IKK);
- Negatively regulate leptin signaling (SOCS3 and PTP1B);
- Inhibit HG-induced caspase-3 activation and apoptosis;
- Increase adiponectin receptor gene expression, upregulate perilipin adipocytes, upregulate PPARγ2, C/EBPα, and activate the adiponectin signaling pathway;
- Regulate PI3K and Akt activity;
- Activate AMPK, regulate energy metabolism, and promote glucose uptake, at least partially, by activating the insulin signaling pathway;
- Regulate NF-κB pathway (p-IKK and p-IκBα) and leptin–STAT3 signaling.

In summary, ginsenoside Rb1 acts as an insulin sensitizer and thus has a hypoglycemic function and this compound may be developed as an anti-obesity, anti-hyperglycemic, and anti-diabetic agent (Figure 2).

## 4. Overview of Anti-Diabetic Complications of Ginsenoside Rb1

Glycolipid metabolism disorder is one of the main physiological characteristics of T2DM, which can result in hyperlipidemia and various microvascular and macrovascular T2DM complications over the long term [87,88]. Therefore, timely and effective glycemic control with diet, sulfonylurea, metformin, or insulin, improvements in glycolipid metabolism, and increases in insulin sensitivity play an irreplaceable role in the treatment of diabetes and the prevention and treatment of complications [88,89]. Based on the current findings, ginsenoside Rb1 can lower glucose, increase insulin sensitivity, and regulate lipid metabolism, while also alleviating the occurrence of T2DM-related complications [36,90], including a progressive decline in β-cell function [84], HG-induced kidney damage or diabetic nephropathy [91], HG-induced nerve damage or diabetic encephalopathy [92,93], and diabetic cardiovascular complications [91,94,95].

### 4.1. Protective Effects on the Islets

Apoptosis is the main form of β-cell death in diabetes. Ginsenoside Rb1 has anti-hyperglycemic effects and can protect pancreatic β-cells by inhibiting apoptosis [84] and hyperglycemia-induced oxidative stress [96]. Ginsenoside Rb1 treatment significantly reduced the percentages of apoptotic cells, nitric oxide (NO) production, and relative inducible nitric oxide synthase (iNOS) expression levels in a HG/cytokine-induced rat pancreatic β-cell line (Rin-m5F) and these effects occurred in addition to the downregulation of iNOS, Bax, Fas, and caspase-3 via the caspase-3 pathway [84], as shown in Table 2.

Furthermore, Rb1 promoted glucagon-like peptide-1 (GLP-1) secretion, and increased the intracellular ATP to ADP ratio concentration and the intracellular Ca^2+^ concentration, as well as significantly increased GLP-1 and upregulated proglucagon; these results indicate that the protective effects on the islets were certainly involved in the enhanced GLP-1 secretion [86], as shown in Table 2. GLP-1 is considered an important incretin that can regulate glucose homeostasis in the gastrointestinal tract after meals. GLP-1 itself has a glucose concentration-dependent hypoglycemic effect and it stimulates islet β-cells to secrete insulin, inhibit food intake, and improve symptoms of diabetes. Hence, ginsenoside Rb1 has the protective effects on islet β-cell via enhancing β-cell viability and promoting GLP-1 secretion.

It is known that abnormal mitochondrial energy metabolism induces reactive oxygen species (ROS) and NO generation by oxidative stress [97]. Hydroxyl radical and hypochlorous acid, two of the strongest ROS, can be significantly and selectively reduced by Rb1 with unique anti-oxidant mechanisms [96]. Therefore, it can be speculated that Rb1 can protect islet or pancreatic β-cells against diabetic injury by inhibiting oxidative stress and apoptosis.

### 4.2. Protective Effects on Diabetic Nephropathy

Glomerular filtration, extracellular matrix hypertrophy, and progressive expansion, which have been considered early features of DN, lead to extra cellular matrix accumulation and glomerular basement membrane thickening, followed by induction of renal fibrosis [98]. Tubulointerstitial fibrosis is a common histological manifestation of chronic kidney disease and the degree of fibrosis is a powerful predictor of glomerular filtration rate decline [98]. Protopanaxadiol and its main representative ingredient, ginsenoside Rb1, prevented the HG-induced increase in fibronectin expression in mesangial cells cultivated under diabetic conditions and this effect was associated with regulating the p44/42, p38, JNK/SAPK of the mitogen activated protein kinase (MAPK) signal pathways, and Akt phosphorylation levels [60], indicating that Rb1 may be used as a remedy for DN, a major complication of diabetes mellitus. Also, the effects of *P. notoginseng* saponins (PNS) and Rb1 were produced partly via reducing the transforming growth factor β1 (TGF-β1) protein expression and blocking the TGF-β1-Smad2/3 signaling pathway [85].

Based on the fact that visceral epithelial cell (podocyte) injury plays a key role in the occurrence and development of DN, the early initiation of therapy that aimed at reducing oxidative stress or modulating ROS-sensitive signaling pathways may be used as a remedy for DN and is considered a new target for the treatment of kidney damage. Simultaneously, ginsenoside Rb1 directly decreases the overproduction of ROS [96], protects against mitochondrial dysfunction [99], and restores the imbalance of cellular redox enzymes [100]. Additionally, Rb1 can reduce excess superoxide anion production and stabilize mitochondrial membrane potential in umbilical vein endothelial cells [101]. Hence, it is speculated that Rb1 may exert protective effects against HG-induced podocyte injury by inhibiting oxidative stress or modulating ROS-sensitive signaling pathways.

### 4.3. Protective Effects on Diabetic Encephalopathy

Patients with DM have a higher risk of developing Alzheimer’s disease (AD) and vascular dementia in the process of aging than non-DM control subjects; this specific disease is recognized as diabetic encephalopathy [102,103]. Metabolic abnormalities, impaired insulin signaling, neuronal apoptosis, oxidative stress, and inflammation are all involved in the development of diabetic encephalopathy (DEP) [48].

As known, ginsenoside Rb1 shows neuroprotective effects [104,105]. It was proven that treatment with Rb1 (1 µM) protected neurons from HG-induced hippocampal neuron injury, effectively inhibited the protein kinase RNA-like endoplasmic reticulum kinase phosphorylation and glycogen synthase kinase3-β expression, and reduced the of C/EBP homologous protein (CHOP) protein levels and mRNA levels (*p* < 0.01) (Table 2), suggesting that ginsenoside Rb1 may protect neurons from HG-induced cell damage by inhibiting glycogen synthase kinase-3β (GSK-3β)-mediated CHOP induction [105]. Moreover, ginsenoside Rb1 exerted neuroprotective effects against oxidative damage and apoptosis in intermittent HG-mediated Schwann cells [63] and reduced the production of ROS, such as 8-hydroxy-2-deoxy guanosine (*p* < 0.01) [96], inhibited intermittent HG-upregulated Bax, and antagonized intermittent HG-downregulated Bcl-2 [63], which provided a potentially new strategy for preventing and treating diabetic encephalopathy.

### 4.4. Protective Effects on Diabetic Cardiovascular Complications

It has been proven that ginsenoside Rb1 can not only inhibit food intake, reduce the diabetic patient’s weight, regulate blood lipids levels, and improve blood hypercoagulability in diabetic patients, but could also delay the occurrence of diabetic heart disease, including microangiopathy, myocardial lesions, and cardiac autonomic neuropathy [94,95], as well as regulate blood pressure in diabetic patients. The significant preventive and therapeutic effects of Rb1 on diabetic cardiovascular complications were summarized and shown in Table 2.

Ginsenoside Rb1 attenuated myocardial ischemia/reperfusion (MI/R) injury in non-diabetic myocardium and in diabetes models [106,107]. Compared to the normal condition, the untreated diabetic condition significantly increased post-ischemic myocardial infarct size, elevated plasma creatine kinase-MB (CK-MB) and lactate dehydrogenase (LDH) release, and reduced blood pressure, accompanied by increased myocardial apoptosis [106]. In contrast, pretreatment of ginsenoside Rb1 reduced myocardial infarct size, decreased plasma CK and LDH, and increased endothelial nitric oxide synthase (eNOS) expression and NO concentration compared with the MI/R group [85]. It also reduced infarct size, cardiomyocyte apoptosis, and caspase-3 activity compared to the diabetic group [107].

Moreover, the preventive and therapeutic effects of Rb1 on diabetic cardiovascular complications was determined and verified in the traditional Chinese herbal medicine and multi-component formula, such as the Shensong Yangxin Capsule. Although this is a traditional compound preparation, its constitution of bioactive components has been initially determined, including ginsenoside Rb1, sodium danshensu, chlorogenic acid, and paeoniflorin. It decreased the heart weight/body weight ratio, attenuated cardiac fibrosis and collagen deposition, and improved the impaired cardiac function of T2DM rats. Meanwhile, it upregulated Smad7, downregulated TGF-β1 and p-Smad 2/3 protein levels and TGF-β1, col-1, col-3, MMP-2, MMP-9, and α-SMA RNA levels, inhibited fibrosis, and improved cardiac function by suppressing TGF-β1/Smad signaling [85].

These effects reveal that ginsenoside Rb1 may exert double-cardioprotective effects on both MI/R injury in diabetes and diabetic damage due to MI/R, partly by inhibiting oxidative stress and apoptosis, enhancing the eNOS expression, and increasing the NO concentration, as well as activating the PI3K/Akt pathway.

### 4.5. Intestine and Gut Microbiota Effects

In addition to the above effects, PNS and ginsenoside Rb1 can modulate the gut microbiota. Ginsenoside Rb1 has significant hypoglycemic activity in the context of diabetes because its gut permeability is increased and its deglycosylation by intestinal microflora is inhibited [57,108]. On the one hand, Rb1 passage through a Caco-2 monolayer was promoted by culturing with diabetic serum and Rb1 oral exposure significantly increased under diabetic conditions; these effects may be associated with the combined contribution of increased gut permeability and inhibited the ginsenoside Rb1 deglycosylation by the intestinal microflora [108]. On the other hand, ginseng polysaccharides and Rb1 reinstated the perturbed holistic gut microbiota and promoted fecal-d-glucosidase activity by modulating the gut microbiota and upregulating the transformation pathway of Rb1 in diabetic rat intestinal microflora (Rb1→Rd→F2→CK) [76].

In addition, ginsenoside Rb1 promoted the secretion of GLP-1, a brain gut peptide secreted by ileal endocrine cells. GLP-1 itself has a glucose concentration-dependent hypoglycemic effect and it stimulates islet β-cells to secrete insulin [109].

Therefore, ginsenoside Rb1 could improve oral bioavailability, modulate gut microbiota composition/balance, increase gut permeability, and stimulate GLP-1 secretion to exert potential hypoglycemic and anti-diabetic effects.

**Figure 3:**
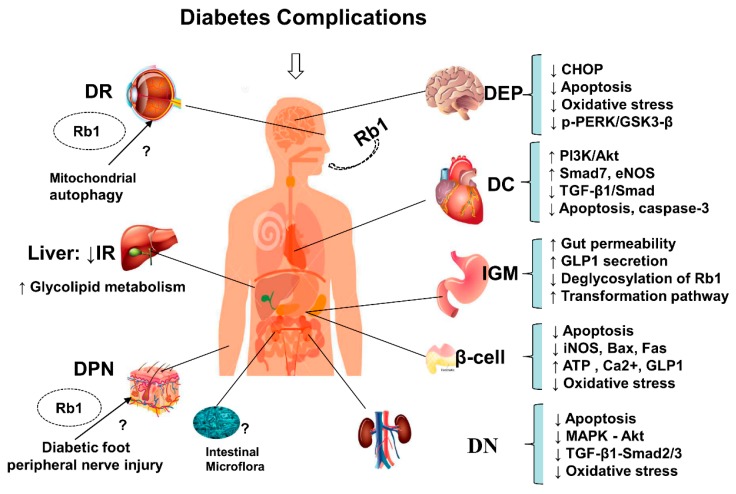
Summary and functional network target analysis of ginsenoside Rb1, which exerts significant anti-hyperglycemic and anti-diabetic effects on diabetes complications via multiple links across regulatory mechanisms and multi-target effects. IR, insulin resistance; DN, diabetic nephropathy; DEP, diabetic encephalopathy; DC, diabetic cardiovascular complications; DR, diabetic retinopathy; DPN, diabetic peripheral neuropathy; IGM, intestine and gut/microbiota. (↓), downregulation or inhibition; (↑), upregulation or activation; (?), uncertainty or undetermined.

In addition, ginsenoside Rb1 not only lowers glucose, increases insulin sensitivity, and regulates lipid metabolism (Figure 2), but also alleviates the occurrence of T2DM-related complications (Figure 3), including the progressive decline in β-cell function, HG-induced kidney damage or DN, HG-induced nerve damage or diabetic encephalopathy, and diabetic cardiovascular complications, as shown in Figure 3.

These effects show the characteristics of multiple targets and joint regulation of multiple pathways as follows:

Rb1 has anti-hyperglycemic effects and protects pancreatic β-cells by inhibiting oxidative stress induced by hyperglycemia. It also reduces cell apoptosis and NO and 8-OHDG production, promotes GLP-1 secretion, enhances islet β-cell viability, increases the ratio of intracellular ATP to ADP concentration, downregulates iNOS expression, and decreases Bax, Fa, and caspase-3 expression via the caspase-3 pathway.

- Rb1 protects neurons from HG-induced cell damage and exerts neuroprotective effects against oxidative damage and apoptosis by inhibiting GSK-3β-mediated CHOP induction and reducing ROS production.
- Rb1 has protective effects against high glucose podocyte injury, kidney damage, and DN by inhibiting oxidative stress. It also decreases ROS overproduction, protects against mitochondrial dysfunction, restores cellular redox enzymes imbalances, and reduces fibronectin expression under diabetic conditions by regulating p44/4, p38-MAPK, JNK/SAPK, and Akt phosphorylation levels, reducing TGF-β1 protein expression, and blocking the TGF-β1-Smad2/3 signaling pathway.
- Rb1 inhibits fibrosis and improves cardiac function by suppressing TGF-β1/Smad signaling. It also exerts cardioprotective effects on both myocardial ischemia/reperfusion under diabetic conditions and diabetic damage from myocardial ischemia/reperfusion. Rb1 attenuates myocardial ischemia/reperfusion injury and reduces infarct size, cardiomyocyte apoptosis, and caspase-3 activity, partly by inhibiting oxidative stress and apoptosis, enhancing eNOS expression, and increasing NO concentrations, as well as activating the PI3K/Akt pathway.

## 5. Conclusion and Recommendation

Diabetes mellitus, a chronic metabolic disease, is a serious problem throughout the world. It is a metabolic disorder characterized by hyperglycemia caused by insufficient insulin secretion and/or insulin action. Type 2 diabetes is the insulin-dependent type and patients must be treated with insulin permanently. The main pathogenesis of T2DM involves islet β-cell dysfunction and peripheral insulin resistance. Insulin resistance is the main characteristic of DM. Currently, the number of DM patients increases each year due to changes in living standards and environment and this number is expected to reach 300 million by 2025 [1,2,3]. After thousands of years of practice, considerable evidence on the use of traditional Chinese medicines and natural medicines for the prevention and treatment of diabetes has accumulated and Chinese medicines and natural medicines have specific curative effects and few side effects. All of these are valuable resources for reference and exploration for the development of new anti-diabetic drugs. Making full use of modern chemical and biological methods to improve our research efforts, it will provide new drugs and ideas for the prevention and treatment of diabetes and its complications [27,28,29,30,31].

Accumulating evidence has shown that ginseng and its main active constituent ginsenoside Rb1 posse anti-diabetic and insulin-sensitizing properties, which may be partly achieved via their ability to regulate adipocyte development and functions. Meanwhile, ginsenoside Rb1 not only has the effects of lowering glucose, increasing insulin sensitivity, and regulating lipid metabolism (Figure 2), but it also alleviates the occurrence of T2DM-related complications (Figure 3), including the progressive β-cell function decline, HG-induced kidney damage, HG-induced nerve damage or diabetic encephalopathy, and diabetic cardiovascular complications, as shown in Figure 3.

In the present review, we summarized the available reports on the therapeutic effects and the molecular mechanisms of ginsenoside Rb1 in diabetes and its complications. Ginsenoside Rb1 exerts significant anti-obesity, anti-hyperglycemic, and anti-diabetic effects by regulating the glycolipid metabolism and improving insulin and leptin sensitivities, as shown in Figure 2. Obviously, ginsenoside Rb1, a promising natural hypoglycemic agent, has a variety of hypoglycemic effects and can effectively control and delay the development of diabetes (Figure 2) and diabetic complications (Figure 3) through scavenging oxygen free radicals, inhibiting protein non-enzymatic glycation, inhibiting aldose reductase, correcting fat and protein metabolism disorders, inhibiting platelet aggregation, increasing insulin sensitivity, and alleviating the occurrence of T2DM-related complications via multiple links across regulatory mechanisms and multi-target effects.

Ginsenoside Rb1, a natural active ingredient, has various pharmacological activities. The current study revealed that Rb1 exerted anti-diabetic effects involved in multiple signaling molecules and multiple pathways, presenting the characteristic of multiple effects, multiple targets, and multiple pathways (Figure 2 and Figure 3). Therefore, the effects and mechanisms of Rb1 in the prevention and treatment of diabetes is comprehensively outlined to illustrate the therapeutic effects of natural active ingredients on systems and complex diseases via multi-pathways, multi-targets and multi-effects, which will provide a therapeutic reference for the treatment of complex metabolic diseases. At present, it is difficult to obtain significant therapeutic effects by treating complex diseases with a single-target strategy. Instead, it provides new ideas and methods with multi-target treatment for such complex diseases. All of these are new perspectives on ginsenoside Rb1 regulating energy metabolism and improving insulin resistance to exert protective effects on diabetes and diabetic complications, which should be further explored.

## Figures and Tables

**Table 1:** Summarized effects and mechanisms of ginsenoside Rb1 on different targets related to diabetes mellitus in animal and in vitro studies.

Model	Type	Inducer	Animal/Cell	Effects	Mechanisms	Ref.
**DM**	In vitro	AdipoR1 sense siRNA	C2C12 myotubes	↑Basal AdipoR levels ↑GLUT4 translocations	↑Translocations of GLUT4; Adiponectin signaling pathway	[61]
**T2DM; Obesity**	In vivo	HFD	Obese mouse	↓Body weight gain ↓Fat accumulation ↑Glucose tolerance	Modulate inflammation ↑Central leptin sensitivity ↓Pro-inflammatory cytokines	[62]
**T2DM**	In vivo	*db/db*	C57; *db/db* mice	↑Insulin sensitivity ↓Adipocyte lipolysis ↓Levels of free fatty acids ↑Hepatic fat accumulation ↓Liver weight, hepatic triglyceride	↓TNF-α ↑Perilipin ↑Insulin sensitivity ↑Level of adiponectin	[64]
**T2DM;** **Obesity**	In vivo	HFD	Rat	↓Food intake ↓Body weight gain ↓Body fat content ↑Energy expenditure	↑c-Fos ↓NPY ↓PI3k/Akt signaling pathway	[71]
**Obesity**	In vivo	KK-Ay	C57 KK-Ay mice	↓Body weight gain ↓FBG and food consumption	↑Insulin/leptin sensitivities ↓Insulin resistance index	[73]
**T2DM; Obesity**	In vitro	Differentiation inducer	3T3-L1 cells	↑Glucose uptake ↑Lipid accumulation ↑Proliferation of 3T3-L1 preadipocytes	↑ap2, GLUT4 ↑Adipogenesis ↑PPARγ2 and C/EBPα	[74]
**T2DM; Obesity**	In vitro	Differentiation inducer	3T3-L1 cells; C2C12 myotubes	↑PI3K activity ↑Glucose uptake ↑IRS1 and PKB phosphorylation	Activating insulin signaling pathway	[75]
**T2DM; Obesity**	In vivo	*db/db*	*db/db* mice	↑GLUT1 and GLUT4 ↑Akt Phosphorylation ↓HOMA-IR and FBG and FINS	↑Glucose metabolism ↑Insulin sensitizing activity	[78]
**T2DM**	In vivo	HFD	C57BL/C mice	↑Glucose tolerance ↓11β-HSD1 levels ↑Fasting blood glucose	↓11β-HSD1 ↑Insulin sensitivity	[79]
**DM**	In vitro	HG; Cytokine	Rin-m5F	↓iNOS expression and NO ↓Pancreatic β-cell apoptosis	↓Caspase-3 ↓Apoptosis-related genes	[84]
**T2DM**	In vivo; In vitro	HFD; STZ	Male SD rats; NCI-H716 cells	↑GLP-1 secretion ↑ATP:ADP ratio	↑GLP-1 ↑Proglucagon	[86]

C/EBPα, recombinant human CCAAT/enhancer binding protein alpha; DM, diabetes mellitus; DC, diabetic cardiomyopathy; DN, diabetic nephropathy; FBG, fasting blood glucose; FINS, fasting insulin; HOMA-IR, homeostasis model assessment-insulin resistance; HG, high glucose; HFD, high-fat diet; GLUT, glucose transporter; HOCl, hypochlorous acid; GLP-1, glucagon-like peptide-1; IL-6 and/or IL-1β, pro-inflammatory cytokines; IRS1, insulin receptor substrate-1; iNOS, inducible nitric oxide synthase; MAPK, mitogen activated protein kinase; MI/R, myocardial ischemia/reperfusion; NO, nitric oxide; PPARγ, peroxisome proliferator-activated receptor γ; PKB/Akt, protein kinase B; PI3K, phosphatidylinositol 3-kinase; Rin-m5F, Rattus pancreatic β-cell line; RF stands for references; STZ, streptozotocin; SD, Sprague–Dawley rat; T2DM, type 2 diabetes; TNF-α, tumor necrosis factor-α; 11β-HSD1, 11β-Hydroxysteroid dehydrogenase type I.

**Table 2:** Summarized effects and mechanisms of ginsenoside Rb1 on different targets related to diabetic complications in animal and in vitro studies, including β-cell injury, kidney damage or diabetic nephropathy, nerve damage or diabetic encephalopathy. and diabetic cardiovascular complications.

Model	Type	Inducer	Animal/Cell	Effects	Mechanisms	Ref.
**T2DM**	In vivo	HFD; HG STZ	Male Wistar rats; Intestinal microflora	↓Blood sugar levels ↑Biotransformation pathway ↑Fecal-d-glucosidase activity	↑Pathway (Rb1→Rd→F2→CK); gut microbiota-mediated bioconversion	[57]
**DM DN**	In vitro	HG	Mesangial cells	↓Phosphorylation of p38, JNK, and p44/42 MAPK ↓Fibronectin expression	↓Phosphorylation of p44/42 MAPK, p38 MAPK, JNK/SAPK, and Akt	[60]
**DPN**	In vitro	HG;	Schwann cells	↓Bax ↓ROS and 8-OHDG	↑Bcl-2 ↓Apoptosis ↓Oxidative stress	[63]
**T2DM**	In vitro	Inducers	3T3-L1 adipocytes C2C12 myotubes	↑PI3K activity ↑Glucose uptake ↑IRS1 and PKB	↑GLUT1 and GLUT4 ↑Insulin signaling pathway	[75]
**DC** **DM**	In vivo	STZ	Diabetic rats	↓Heart weight/body weight ↑Impaired cardiac function ↓Col-1, col-3, MMP-2, MMP-9 and α-SMA	↑Smad7 ↓Cardiac fibrosis ↓TGF-β1 and p-Smad2/3, TGF-β1/Smad signaling	[85]
**DM**	In vitro	HG; cytokine	Rin-m5F	↓NO production ↓Pancreatic β-cell apoptosis	↓iNOS ↓Fas and caspase-3	[84]
**T2DM**	In vivo; In vitro	HFD; STZ	Male SD rats NCI-H716 cells	↑Ratio of the ATP:ADP ↑GLP-1 secretion	↑GLP-1 ↑Proglucagon	[86]
**DM**	In vitro	ROS	Cell-free system	↓·OH and HOCl	Unique anti-oxidant mechanisms	[96].
**DM**	In vitro	HG	Hippocampal neurons	↓Neuronal loss ↑Cell viability	↓CHOP protein ↓p-PERK and p-GSK-3β	[105]
**DM** **MI/R**	In vivo	STZ; MI/R	Male SD rat	↓Plasma CK and LDH ↓Myocardial infarct size ↓Myocardial oxidative stress	↑eNOS and NO; ↓Oxidative stress	[106]
**DM** **MI/R**	In vivo	STZ; MI/R	Male SD rat	↓Infarct size ↓Caspase-3 activity ↓Cardiomyocyte apoptosis	↑Phosphorylated Akt; activation of PI3K/Akt pathway	[107]
**T2DM**	In vivo; In vitro	HFD; STZ	Male SD rat Caco-2 cells	↑Rb1 absorption ↑Rb1 systemic exposures ↑Portal Rb1 concentration	↓Rb1 deglycosylation ↑Rb1 intestinal absorption	[108]

CK, Creatine Kinase; DPN, diabetic peripheral neuropathy; DM, diabetes mellitus; DN, diabetic nephropathy; eNOS, endothelial nitric oxide synthase; GSK-3β, glycogen synthase kinase-3β; GLUT, glucose transporter; GLP-1, glucagon-like peptide-1; HG, high glucose; HFD, high-fat diet; HOCl, hypochlorous acid; IRS1, insulin receptor substrate-1; LDH, lactate dehydrogenase; MI/R, myocardial ischemia/reperfusion; MAPK, mitogen activated protein kinase; NO, nitric oxide; PERK, protein kinase RNA-like ER kinase; PKB/Akt, protein kinase B; PI3K, phosphatidylinositol 3-kinase; ·OH, hydroxyl radical; Rin-m5F, Rattus pancreatic β-cell line; STZ, streptozotocin; SD, Sprague–Dawley rat; RF stands for references; TGF-β1, transforming growth factor β1; T2DM, type 2 diabetes; 8-OHDG, 8-hydroxy-2-deoxy deoxyguanosine.
